# Experimental Study on the Performance of Light-Controlled Ion Drag Pump Based on PLZT Ceramic

**DOI:** 10.3390/mi17010045

**Published:** 2025-12-29

**Authors:** Yujuan Tang, Yujie Shi, Zhen Lv, Zihao Guo, Xinjie Wang

**Affiliations:** 1 School of Intelligent Science and Control Engineering, Jinling Institute of Technology, Nanjing 211169, China; zhguo020528@163.com; 2 School of Mechanical Engineering, Nanjing University of Science and Technology, Nanjing 210094, China; yjshi@njust.edu.cn (Y.S.); zlv@njust.edu.cn (Z.L.); xjwang@njust.edu.cn (X.W.)

**Keywords:** electrohydrodynamics, PLZT ceramics, charge injection, ion drag pump, pumping performance

## Abstract

Light-controlled ion drag pumps have attracted considerable interest in soft robotics, biomedical engineering, and microelectromechanical systems (MEMS) due to their non-contact energy supply and high spatiotemporal controllability of light. However, experimental studies on their pumping performance and influencing factors remain limited. This study integrates the photoelectric effect with field emission phenomena to design and fabricate a light-controlled ion drag pump using lanthanum-modified lead zirconate titanate (PLZT) ceramic. The light-controlled pump enables non-contact energy transfer and fluid transport via high-energy laser irradiation. A series of experiments systematically investigate its pumping performance and key influencing factors. Results indicate that optimizing electrode structure and fluid channel design, along with increased light intensity, significantly enhances pumping performance. This work provides fundamental design guidelines for the application of light-controlled ion drag pumps in microfluidics, flexible robotics, and microdevice thermal management.

## 1. Introduction

The advancement of micro-electro-mechanical systems (MEMS) and micro-optical-electro-mechanical systems (MOEMS) has created a strong demand for compact, efficient, and wireless micro-actuation technologies [1]. Conventional micro-pumps, driven by electrostatic, piezoelectric, electrothermal, or electromagnetic principles, often involve moving parts, complex wiring, and susceptibility to electromagnetic interference [2]. In contrast, electrohydrodynamic (EHD) pumps, which operate on Coulomb forces without moving parts, offer advantages such as silent operation, simplicity, and high reliability [3]. Among EHD methods, ion drag pumps in which charges injected into a dielectric fluid drag the fluid from an emitter to a collector electrode show broad application potential in fluid actuation, particularly in microfluidics, soft robotics, and microdevice cooling [4,5].

However, a significant limitation persists: Ion drag pumps typically require an external high-voltage power supply (often kilovolts) [6,7], which complicates system integration, increases size, and generates electromagnetic noise, thereby hindering miniaturization and use in sensitive environments. Recent research has focused on optimizing electrode configurations [8], adopting interdigitated electrodes [9,10] to improve the performance of the ion drag pumps, but the fundamental reliance on wired, high-voltage sources remains unaddressed. The integration of high-voltage power supply units complicates the overall pumping system and is prone to generating electromagnetic interference, which hinders the development of injection pump systems in terms of miniaturization, high integration, and electro-magnetic interference resistance.

Parallelly, lanthanum-modified lead zirconate titanate (PLZT) ceramics represent a distinctive photoelectric smart material. Under ultraviolet (UV) excitation, they exhibit anomalous photovoltaic, thermoelectric, photothermal, thermal expansion, and piezoelectric effects [11]. From a photophysical perspective, the kilovolt-level photovoltage generated when UV light irradiates the PLZT polarization direction is highly significant for practical applications, e.g., hybrid photo-vibrational energy harvesting and micro-drive. Compared with conventional solar cells, which typically generate low-voltage direct current requiring power conditioning circuits for most applications, PLZT ceramics can produce a high photovoltage (on the order of kilovolts) directly, eliminating the need for external voltage amplifiers. Their operation generates minimal electromagnetic interference, making them suitable for noise-sensitive environments such as biomedical or precision measurement systems. This property has been exploited in lunar dust removal devices [12], novel actuators [13], and micro gripper [14] and damping systems [15], suggesting great potential for clean, light-driven micro-systems. Thus, the rapid photovoltage generated by PLZT ceramics under illumination can provide energy for injection pumps, thereby enabling light-controlled pumping of fluids.

In contrast to previous ion drag pumps that rely on external high-voltage supplies, this work introduces a fundamentally distinct driving strategy by integrating the photoelectric effect of PLZT ceramics with charge injection pumping. We present, for the first time, a fully light-controlled ion drag pump where the high photovoltage generated by PLZT ceramics under UV illumination directly powers the pump electrodes, enabling wireless and non-contact fluid actuation. Beyond the conceptual integration, this study provides a systematic experimental investigation into the key performance-influencing factors (light intensity, electrode geometry, and channel dimensions), establishing practical design guidelines. This approach not only addresses the miniaturization and electro-magnetic interference challenges associated with traditional high-voltage sources but also opens new avenues for optically addressable microfluidics and soft robotic systems.

## 2. Mathematical Modelling of the Light-Controlled Ion Drag Pump Based on PLZT Ceramic

**Figure 1:**
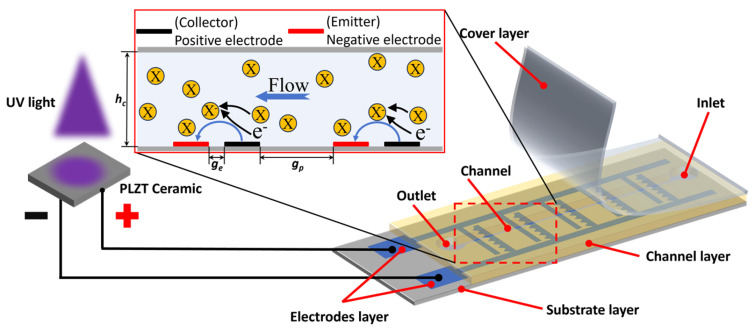
Structure and working mechanism of the light-controlled ion drag pump based on PLZT ceramic.

Figure 1 illustrates the schematic of the light-controlled PLZT ion drag pump. The system comprises a PLZT ceramic providing high voltage and an ion drag pump chip, interconnected via wires. The pump chip features a four-layer sandwich structure (substrate, electrode, channel, and cover layers). The staggered electrode structure within the electrode layer generates the required electric field upon receiving voltage from the PLZT ceramic, driving dielectric fluid flow in the channel layer. A single-sided serrated electrode structure is adopted for the pump chip. To prevent dielectric breakdown, dielectric materials are used for the substrate, channel, and cover layers. In this design, UV light (wavelength ~365 nm) irradiates the PLZT ceramic, inducing a rapidly increasing photovoltage via the photoelectric effect. This photovoltage is applied to the electrode layer of the pump chip. As shown in Figure 1, the pump chip’s positive and negative electrodes function as the collector and emitter, respectively. When the photoelectric field strength surpasses the energy barrier, field emission occurs, injecting electrons from the emitter into the dielectric fluid. Neutral molecules capture these electrons, forming ions. Under the photoelectric field, ions migrate toward the collector, releasing electrons and thereby driving fluid flow. Upon UV cessation, the PLZT ceases photovoltage generation, the inter-electrode field gradually dissipates, and flow stops. Thus, powered solely by PLZT-generated photovoltage, this pump operates without external power sources, offering significant miniaturization advantages. Key dimensional parameters of the pump chip are labeled in Figure 1. For the ion drag pump chip, the channel height *h_c_*, electrode spacing *g_e_*, and gap between the electrode pairs *g_p_*, are the primary design variables investigated in this study. Their values are varied within the range of 0.3 mm to 2 mm in subsequent experiments to systematically evaluate their influence on pumping performance.

According to related literature, PLZT ceramics can be represented as an equivalent circuit consisting of a current source *I_p_* in parallel with a resistor *R_p_* and a capacitor *C_p_*. When PLZT ceramics are connected to the ion drag pump, the equivalent capacitance and resistance of the pump are effectively placed in parallel with resistor *R_p_* and a capacitor *C_p_* in parallel. The photovoltage *V_p_* of the light-controlled ion drag pump is expressed as follows [15]:

(1) $$V_{p}=I_{p}R_{eq}\left(1-e^{-\frac{t}{\tau }}\right)$$

where *R_eq_* is the equivalent resistance of the light-controlled ion drag pump $R_{eq}=\frac{R_{p}R_{pump}}{R_{p}+R_{pump}}$, and *τ* is the time constant $\tau =\frac{\left(C_{p}+C_{pump}\right)R_{p}R_{pump}}{R_{p}+R_{pump}}$; and *C_pump_* and *R_pump_* represent the equivalent capacitance and resistance of the pump.

Additionally, according to the Korteweg–Helmholtz formula, the electric volume force exerted on a unit volume of fluid in an electric field *F_d_* is:

(2) $$F_{d}=\rho_{d}E-\frac{1}{2}E^{2}\nabla \varepsilon +\frac{1}{2}\nabla \left[E^{2}\rho {\left(\frac{\partial \varepsilon }{\partial \rho }\right)}_{T}\right]$$

where *ρ_d_*, *E*, *ε*, and *ρ* denote the space charge density, electric field strength, dielectric constant of the fluid, and density of the fluid, respectively.

Equation (2) comprises three components: the Coulomb force (electrophoretic term) acting on free charges, the dielectrophoretic force arising from permittivity gradients, and the electrocapillary force relevant in compressible dielectrics. For an incompressible fluid, the third term vanishes.

Based on the law of conservation of mass, the continuity equation for fluids can be derived:

(3) $$\frac{\partial \rho }{\partial t}+\nabla \cdot \rho v=0$$

where *t* is time and *v* is the flow velocity.

Equation (3) rests on the continuum assumption, which is valid for the flow regimes considered in this EHD analysis. According to the principle of conservation of momentum, fluid motion in a flow field satisfies the Navier–Stokes momentum equation:

(4) $$\rho \left[\frac{\partial u}{\partial t}+(v\cdot \nabla )v\right]=-\nabla p+\eta \nabla^{2}v+F_{d}$$

where *η* and *p* are the fluid viscosity and the pressure, respectively.

According to the electrostatic Poisson’s equation, the relationship between the electric field and photovoltage is:

(5) $$E=-\nabla V=-\nabla \left[I_{p}\left(\frac{R_{p}R_{pump}}{R_{p}+R_{pump}}\right)\left(1-e^{-\frac{t}{\left(C_{p}+C_{pump}\right)R_{p}R_{pump}}\left(R_{p}+R_{pump}\right)}\right)\right]$$

The space charge density *ρ_d_* follows charge conservation:

(6) $$\frac{\partial \rho_{d}}{\partial t}+\nabla \cdot J=0$$

where *J* is current density, representing total charge passing per unit area per unit time. Current density *J* is expressed as:

(7) $$J=\mu_{d}\rho_{d}E+D\nabla \rho_{d}+\rho_{d}u+\sigma_{d}E$$

where *μ_d_*, *D*, and *σ_d_* represent the electron mobility coefficient, molecular diffusion coefficient, and electrical conductivity coefficient, respectively.

Additionally, the output performance of this light-controlled micro-injection pump is defined by its output pressure and output flow rate. The output pressure can be calculated using Equation (5), while the flow rate *Q* is obtained by integrating the velocity profile over the channel cross-section *A*:

(8) $$Q=\int_{A}vdA$$

## 3. Experimental Analysis of Influencing Factors on the Output Performance of the Light-Controlled Ion Drag Pump

### 3.1. Experimental Platform for the Output Performance of Light-Controlled PLZT Ion Drag Pump

**Figure 2:**
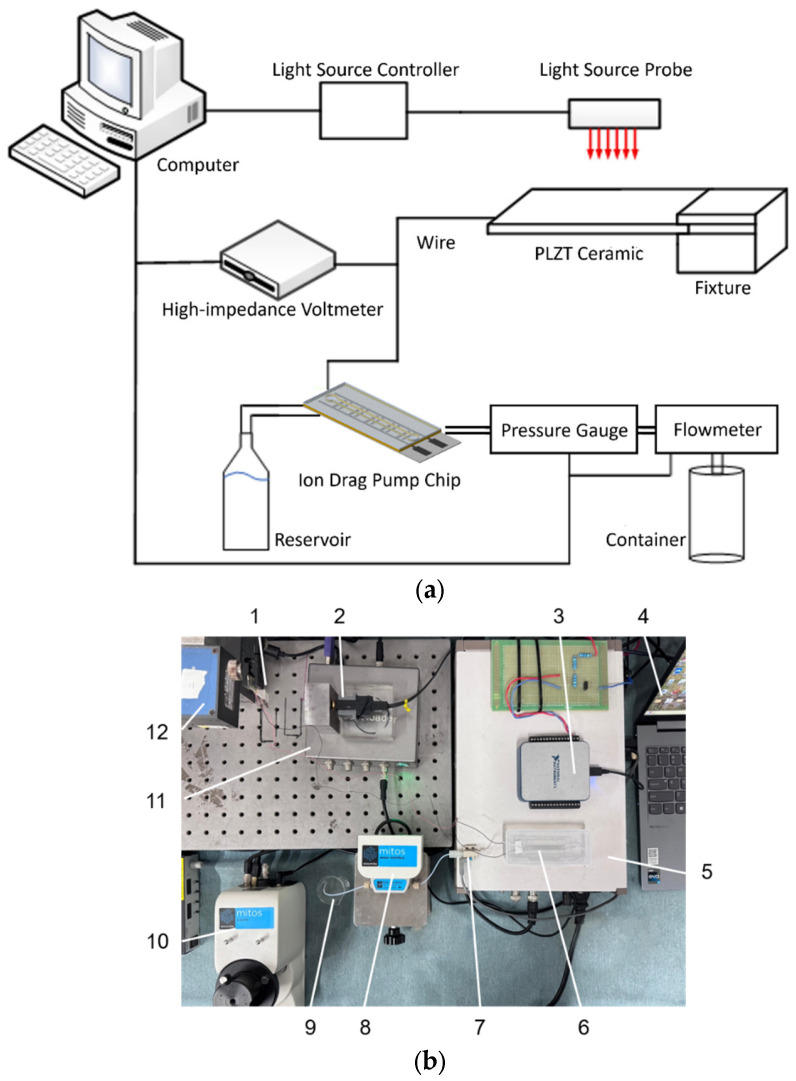
Experimental platform of the performance of light-controlled ion drag pump: (**a**) Schematic diagram; (**b**) Experimental platform: 1—PLZT ceramic, 2—high-impedance voltmeter probe, 3—data acquisition card, 4—computer, 5—high-impedance voltmeter, 6—micro-injection pump chip, 7—pressure gauge, 8—flow meter, 9—beaker, 10—flow meter controller, 11—pressure gauge controller, and 12—continuous-wave UV light probe.

Figure 2 shows the experimental platform for evaluating the output performance of the light-controlled ion drag pump. During testing, the PLZT ceramic is continuously irradiated with adjustable-intensity continuous-wave UV light. A pressure gauge (PS4, Shanghai Pengzan Biotechnology Co., Ltd., Shanghai, China) and a flowmeter (MicroFluidics, Dolymate Ltd., Royston, UK) measure output pressure and flow rate, respectively. Illumination duration is 300 s, with data collection continuing for 400 s post-illumination. The cantilever configuration of the PLZT ceramic enables the UV light to be easily aligned vertically with the top surface of the PLZT ceramic and reduces parasitic capacitive coupling and unintended charge leakage paths of the photo-induced voltage. A standard ion drag pump chip (channel height: 1 mm, electrode spacing: 0.3 mm, gap between electrode pairs: 0.6 mm, 25 electrode pairs) and a PLZT ceramic patch (15 mm × 5 mm × 1 mm) are used in this paper.

**Figure 3:**
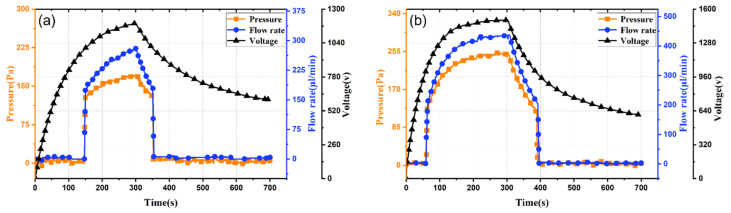
Dynamic response curves of the light-controlled ion drag pumps under different light intensities: (**a**) 50 mW/cm^2^; (**b**) 100 mW/cm^2^.

PLZT ceramics generate different photovoltages under varying UV intensities. To characterize pump performance under different light intensities, pressure and flow rate are tested at 50 mW/cm^2^ and 100 mW/cm^2^, as shown in Figure 3. Comparison of Figure 3a,b shows that increasing light intensity from 50 mW/cm^2^ to 100 mW/cm^2^ shortens the pump’s response time from 150 s to 53 s. The difference in response time is fundamentally governed by the photo-charging dynamics of the PLZT ceramic. As described by Equation (1), the photovoltage *Vp* builds up over time upon illumination. A higher light intensity (100 mW/cm^2^) leads to a higher photovoltage *Vp* and smaller time constant, which in turn charges the equivalent circuit (Rp//Cp) more rapidly. Consequently, the threshold electric field required for charge injection and pump activation is reached in a shorter time (~70 s) compared to the lower intensity case (50 mW/cm^2^, ~150 s). Peak output pressure and flow rate increase from 0.17 kPa and 280 μL/min to 0.25 kPa and 430 μL/min, representing increases of 47% and 53%, respectively. These results validate the performance and feasibility of the light-controlled ion drag pump. Furthermore, the performance of the pump can be adjusted by changing the light intensity.

Subsequent sections investigate key factors affecting output performance: microchannel height, electrode spacing, number of electrode pairs, and gap between electrode pairs. Experimental data are compared with numerical simulations based on the mathematical model.

### 3.2. Effect of Microchannel Height

**Figure 4:**
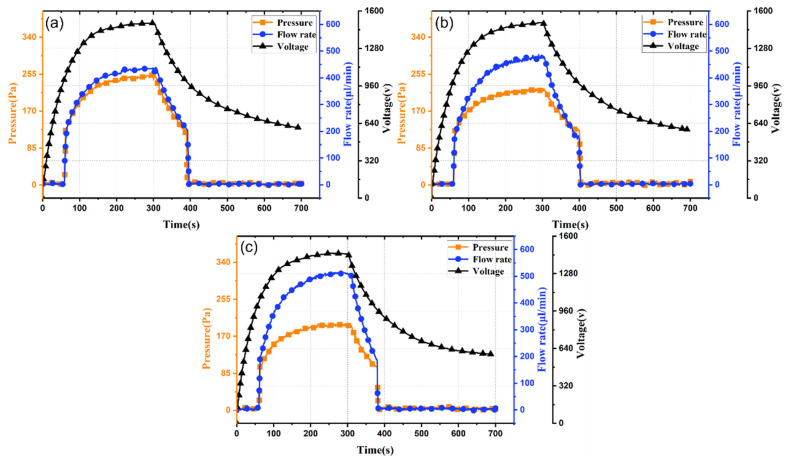
Time-dependent performance curves of light-controlled ion drag pumps at different channel heights: (**a**) 1 mm; (**b**) 1.5 mm; and (**c**) 2 mm.

Figure 4 presents experimental results for light-controlled ion drag pumps with channel heights of 1 mm, 1.5 mm, and 2 mm. As shown in Figure 4a–c, channel height has negligible impact on output response time, which remains about 55 s for all heights, determined by the PLZT photovoltaic voltage characteristics, which are unchanged across channel heights. Thus, channel height does not affect response speed.

Comparing output pressure and flow rate reveals that as channel height increases, peak output pressure decreases, while output flow rate increases. At 100 mW/cm^2^, increasing channel height from 1 mm to 2 mm reduces maximum output pressure from 250 Pa to 200 Pa, while maximum flow rate increases from 430 μL/min to 510 μL/min (18.6% increase). Therefore, increasing channel height effectively enhances output flow rate but reduces output pressure.

The inverse relationship between the channel height and the output pressure, as well as the positive impact on the flow rate, is consistent with the basic principles of fluid dynamics. A larger cross-sectional area reduces the flow resistance, thereby achieving a higher volumetric flow rate. However, for a given charge injection density and electric field strength, the momentum transferred by the unit volume of fluid decreases as the channel expands, resulting in a lower achievable pressure. This trade-off between pressure and flow rate is crucial for adjusting the pump according to specific application requirements, such as seeking a higher flow rate for cooling or a higher pressure to overcome system resistance.

To validate the mathematical model from Section 2, Table 1 compares simulated and experimental maximum output pressures for different channel heights at 100 mW/cm^2^. The close agreement confirms model reliability.

### 3.3. Effect of Electrode Spacing

**Figure 5:**
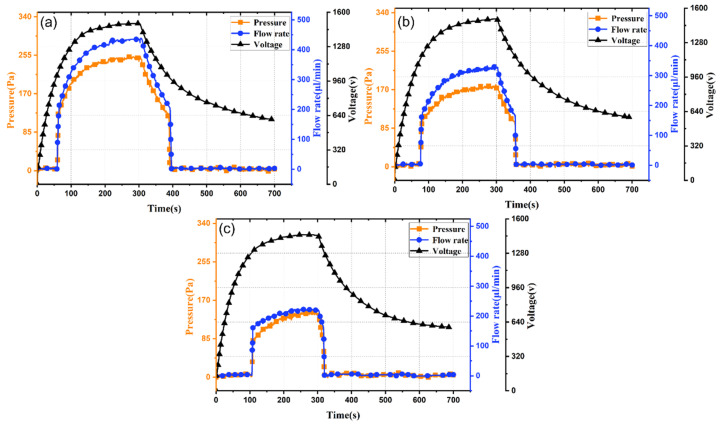
Time-dependent output performance curves of light-controlled ion drag pumps with different electrode spacings: (**a**) 0.3 mm; (**b**) 0.4 mm; and (**c**) 0.5 mm.

Electric field strength distribution, crucial for fluid driving capability, is significantly influenced by positive–negative electrode spacing. Experiments investigated pumps with electrode spacings of 0.3 mm, 0.4 mm, and 0.5 mm. Figure 5 shows time-dependent performance curves of the light-controlled ion drag pumps.

Comparing Figure 5a–c, increasing electrode spacing from 0.3 mm to 0.5 mm prolongs output response time from 57 s to 109 s. This is attributed to differing times to reach the threshold activation electric field: smaller spacing yields higher field strength under identical voltage, enabling faster response. Moreover, increasing electrode spacing from 0.3 mm to 0.5 mm reduces peak output pressure and flow rate from 0.25 kPa and 430 μL/min to 0.14 kPa and 220 μL/min (decreases of 78% and 95%, respectively). Thus, reducing electrode spacing effectively enhances output flow rate, pressure, and response speed.

The significant sensitivity of pump performance to electrode spacing highlights the crucial role of electric field intensity in controlling the ion drag mechanism. As the electrode spacing increases, the response time significantly increases, which underscores the key limitations and design insights of the light-controlled ion drag pump: the activation of the pump depends on the photovoltage reaching the threshold electric field intensity. A larger electrode spacing requires a higher absolute voltage to reach this threshold, thereby prolonging the charging time of the equivalent RC circuit of the PLZT pump. This result strongly indicates that reducing the electrode size is the main strategy for achieving faster activation of light-driven EHD pumps.

Table 2 compares simulated and experimental maximum output pressures for different electrode spacings. Errors are within 7.1%.

### 3.4. Effect of Number of Electrode Pairs

**Figure 6:**
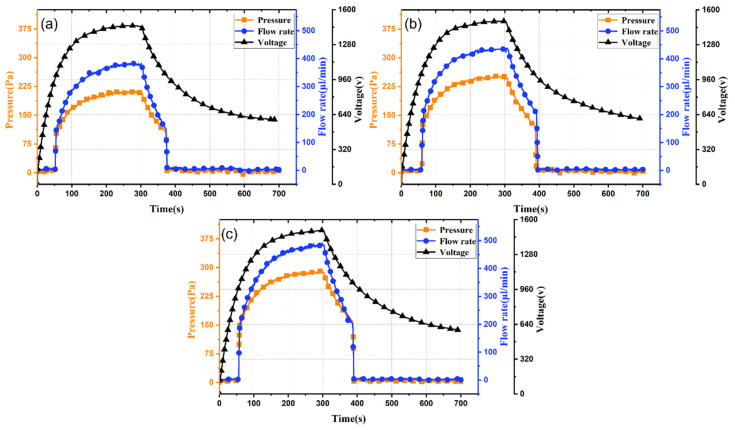
Time-dependent output performance curves of light-controlled ion drag pumps with different numbers of electrode pairs: (**a**) 20 pairs; (**b**) 25 pairs; and (**c**) 30 pairs.

The effect of the number of planar interdigitated electrode pairs on output performance is investigated using pumps with 20, 25, and 30 pairs, as shown in Figure 6. Figure 6 shows that as the number of electrode pairs increases, output response time remains nearly constant (~57 s), with response voltage around 100 V. However, increasing electrode pairs from 20 to 30 raises output pressure amplitude from 0.21 kPa to 0.29 kPa and flow rate from 380 μL/min to 490 μL/min (increases of 38% and 29%, respectively). Thus, increasing electrode pairs effectively enhances maximum output pressure and flow rate.

The linear enhancement of output performance with an increasing number of electrode pairs demonstrates the effective summation of electrohydrodynamic forces along the flow path. Each additional electrode pair gradually increases the contribution to the total pressure head, as it expands the area where the electric volume force is applied. Under different numbers of electrode pairs, the response time of the light-controlled pump remains almost unchanged, which is an important finding. It indicates that the excitation dynamics are controlled by the local field conditions at the first few electrode pairs rather than the total system capacitance. Once the threshold field is reached locally, pumping will almost simultaneously start throughout the channel.

Table 3 compares simulated and experimental maximum output pressures for different numbers of electrode pairs. Errors are within 7.6%.

### 3.5. Effect of Gap Between the Electrode Pairs

**Figure 7:**
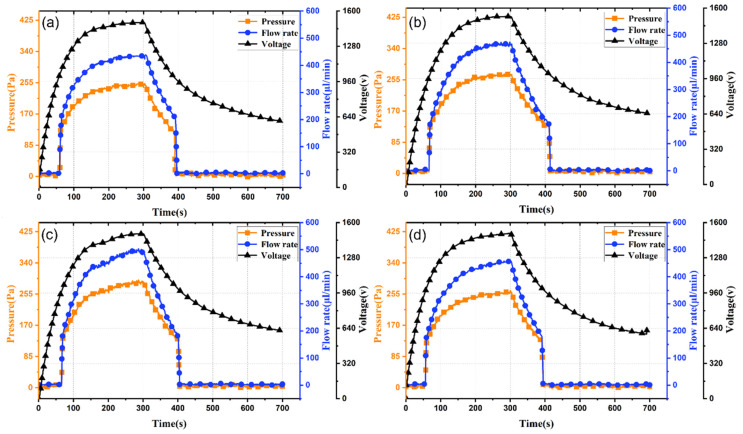
Time-dependent output performance curves of light-controlled ion drag pumps with different gaps between the electrode pairs: (**a**) 0.6 mm; (**b**) 0.8 mm; (**c**) 1 mm; and (**d**) 1.2 mm.

The operating mechanism of the light-controlled ion drag pump involves an opposing electric field between adjacent electrode pairs, counteracting the driving field and impeding fluid flow. To investigate the effect of the electrode pair gap, experiments were conducted with gaps of 0.6 mm, 0.8 mm, 1 mm, and 1.2 mm. Results are shown in Figure 7.

Varying the gap between electrode pairs has negligible effect on output response time, which remains about 55 s, determined by the PLZT photovoltaic voltage characteristics. Comparing output flow rate and pressure across gaps (Figure 7) shows that as the gap increases from 0.6 mm to 1.2 mm in 0.2 mm increments, both output pressure and flow rate initially rise then fall. Increasing the gap reduces the opposing electric field strength, enhancing output performance to some extent. However, further gap increase diminishes fluid kinetic energy transfer along the channel and, with fixed electrode pairs, lengthens the channel, increasing friction losses. Consequently, overall output performance decreases. Experimentally, optimal output performance occurs at a 1 mm gap, with maximum output pressure of 300 Pa and flow rate of 510 μL/min. Thus, rational gap selection effectively improves output performance.

In terms of output performance, the non-monotonic trend observed with the change in the gap between adjacent electrodes reveals the complex interplay between competitive electrical effects and flow effects. There exists an optimal gap (in this study, 1 mm), which can balance the two factors: (1) A larger gap will weaken the undesired reverse electric field between adjacent electrodes of the same polarity, thereby allowing for more effective net positive pumping. (2) When the gap is excessively large, the flow force generated by one electrode pair will decay before reaching the next electrode pair, thereby reducing the effective coupling of forces. Moreover, for a fixed number of electrode pairs, a longer total channel for a fixed number of pairs increases frictional losses. The existence of this optimal gap highlights the importance of the overall design, which requires considering the spatial distribution of the electric field and the resulting flow field morphology, rather than focusing on a single geometric parameter.

Table 4 compares simulated and experimental maximum output pressures for different gaps between electrode pairs. Errors are within 6.2%.

### 3.6. Stall Pressure Test of the Light-Controlled Ion Drag Pump

**Figure 8:**
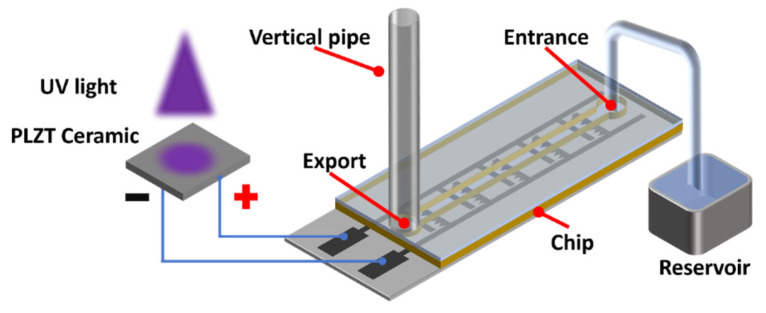
Schematic diagram of stall pressure test of the light-controlled ion drag pump.

**Figure 9:**
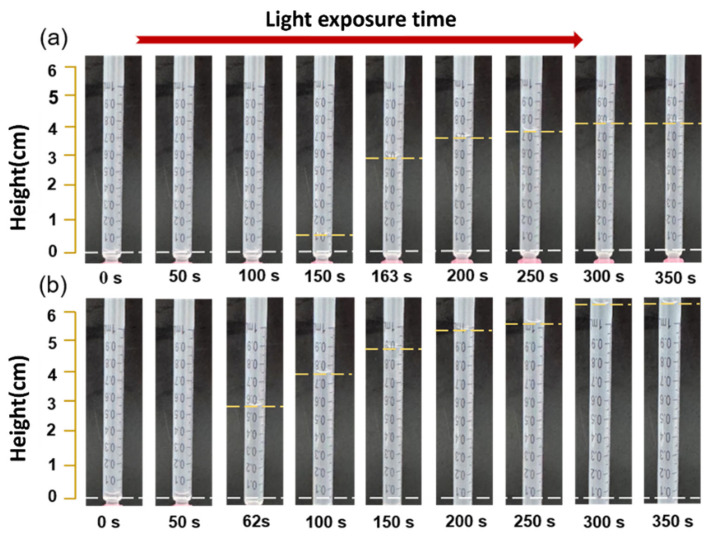
Stall pressure of the light-controlled ion drag pump under different light intensities: (**a**) 50 mW/cm^2^; (**b**) 100 mW/cm^2^.

Considering that fluid pumps must overcome hydraulic loads present in fluid flow during practical applications, such as flow resistance in channels, micro pumps require a certain load-driving capability. The metric for evaluating a fluid micro pump’s load-driving capability is stall pressure, defined as the maximum output pressure achievable when the pump’s output flow rate is zero. Figure 8 presents a schematic diagram of the load capacity testing platform for the light-controlled PLZT ion drag pump. Select the optimal set of parameters from the aforementioned study—namely an ion drag pump chip with a channel height of 1 mm, electrode spacing of 0.3 mm, electrode pair spacing of 1 mm, and 30 electrode pairs—for experimentation. Figure 9 illustrates the temporal variation in liquid level height at the pump outlet channel under different light intensities, revealing the static response characteristics of the ion drag pump. During the initial phase, when illumination begins, the photovoltaic voltage has not yet reached the threshold activation electric field of the ion drag pump, causing the outlet liquid level height to remain unchanged. As the photovoltaic voltage reached and exceeded the threshold activation electric field while continuing to increase, the liquid level height also rose continuously. After illumination ceased, the photovoltaic voltage began to decrease. When the internal photovoltaic electric field of the ion drag pump fell below the threshold activation electric field, its static output pressure dropped to zero. As shown in the experimental results of Figure 9, the liquid level inside the test tube continued to rise with prolonged irradiation time.

In practical applications, fluid pumps must overcome hydraulic loads such as flow resistance. Stall pressure, the maximum output pressure at zero flow rate, evaluates a micro pump’s load-driving capability. Figure 8 shows the stall pressure testing platform schematic. The optimal parameter set (channel height: 1 mm, electrode spacing: 0.3 mm, electrode pair gap: 1 mm, 30 electrode pairs) is selected. Figure 9 shows the temporal variation of liquid level height at the pump outlet under different light intensities, revealing static response characteristics. Initially, upon illumination, before the photovoltaic voltage reaches the threshold activation electric field, the outlet liquid level remains unchanged. As the voltage exceeds and continues to increase, the liquid level rises. When the photovoltage reaches saturation, the liquid level remains stable. Figure 9 shows the liquid level rising with prolonged irradiation. We used the pressure formula:

(9) $$p=\rho_{d}gh$$

where *h* is the liquid length in the vertical pipe.

At 50 mW/cm^2^ and 100 mW/cm^2^, the stall pressures of the light-controlled pump are about 0.7 kPa and 1 kPa, respectively.

## 4. Conclusions

This study presents a light-controlled ion drag pump based on PLZT ceramic by integrating the photoelectric effect with field emission. An EHD model for the pump was developed. Experimental studies on the photoelectric response characteristics of PLZT ceramics integrated into ion drag pump chips established a foundation for their application. An experimental platform was constructed to investigate key factors influencing output performance, including electrode shape, channel height, electrode spacing, number of electrode pairs, and gaps between electrode pairs. Under constant light intensity, output pressure and flow rate were measured to compare the effects of fluid channel dimensions and electrode structure sizes. Experimental results show that fluid channel height, number of electrode pairs, and electrode spacing monotonically affect output performance: Output performance is proportional to channel height and number of electrode pairs, and inversely proportional to electrode spacing. Notably, output pressure and flow rate first increase then decrease as the gap between electrode pairs widens.

This work establishes a basic framework for the light-controlled ion drag pump and verified its viability through systematic experimentation. The unique driving mode—utilizing light to both power and control the pump—opens up unique application potential that is different from the limitations faced by traditional wired, high-voltage charge, gradient-driven pumps. Specifically, this pump is particularly suitable for scenarios that require wireless, low electromagnetic interference, and spatially selective liquid driving. Promising application areas include:

(1) Optically Addressable Microfluidic Networks: In integrated lab-on-a-chip systems, different pump units on a single substrate can be independently activated by focused light beams, enabling complex and reconfigurable control of fluid paths without the need for a matrix structure composed of physical wires or connectors. This facilitates highly multiplexed and programmable biological detection.

(2) Closed-Environment or Implantable Devices: For systems where electrical conduction is not allowed or is not suitable (such as sealed containers used for sensitive chemical reactions, or possible future implantable drug delivery systems), light becomes a clean and safe medium for the transmission of energy and control signals.

(3) Microfluidics in Electromagnetically Sensitive Environments: For operations where the complete absence of generated electromagnetic interference is critical. This includes the microfluidic preparation, processing, or online detection of flammable chemicals and explosive materials, or operations carried out in systems integrated with high-precision electromagnetic sensors. By eliminating the arc discharge and broadband electronic noise inherent in traditional high-voltage drives, it offers a fundamentally safer and more compatible driving solution for these high-reliability and safety-critical applications.

(4) Distributed Drive in Soft Robotics: As a wireless pressure source, it can be embedded in soft robotic structures. Selective illumination of different pump units allows for localized, model-free control of fluidic channels, thereby achieving bending or shape deformation. This simplifies the design and control architecture of the robot.

Future efforts aimed at significantly reducing the response time and further miniaturizing the system are crucial for translating these conceptual advantages into practical applications. The current performance parameters (e.g., response time on the order of tens of seconds, pressure output of ~1 kPa) have already made it possible for application in microfluidics and soft robotics, where speed is not the main limiting factor and wireless control is of critical importance.

## Figures and Tables

**Table 1:** Comparison of numerical simulation and experimental results for the maximum output pressure at different channel heights.

	1 mm	1.5 mm	2 mm
**Simulation (Pa)**	265 Pa	236 Pa	213 Pa
**Experiment (Pa)**	250 Pa	222 Pa	200 Pa

**Table 2:** Comparison of numerical simulation and experimental results for the maximum output pressure at different electrode spacings.

	0.3 mm	0.4 mm	0.5 mm
**Simulation (Pa)**	265	182	149
**Experiment (Pa)**	250	170	140

**Table 3:** Comparison of numerical simulation and experimental results for the maximum output pressure with different numbers of electrode pairs.

	20 pairs	25 pairs	30 pairs
**Simulation (Pa)**	225	265	308
**Experiment (Pa)**	210	250	290

**Table 4:** Comparison of numerical simulation and experimental results for the maximum output pressure with different gaps between the electrode pairs.

	0.6 mm	0.8 mm	1 mm	1.2 mm
**Simulation (Pa)**	265	288	318	270
**Experiment (Pa)**	250	272	300	255

## Data Availability

All data are presented within the paper.
